# Lessons from the finish line: understanding cardiac arrest in endurance racing

**DOI:** 10.1093/europace/euaf321

**Published:** 2025-12-17

**Authors:** Tobias Skjelbred, Sanjay Sharma, Jacob Tfelt-Hansen

**Affiliations:** The Department of Cardiology, The Heart Centre, Copenhagen University Hospital Rigshospitalet, Section 2142, Blegdamsvej 9, Copenhagen 2100, Denmark; Cardiovascular and Genetic Research Institute, City St. George’s, University of London, London, UK; The Department of Cardiology, The Heart Centre, Copenhagen University Hospital Rigshospitalet, Section 2142, Blegdamsvej 9, Copenhagen 2100, Denmark; Department of Forensic Medicine, Section of Forensic Genetics, Faculty of Health and Medical Sciences, University of Copenhagen, Copenhagen, Denmark

**This editorial refers to ‘Characteristics of sudden cardiac arrest during endurance racing: a decade of the Paris registry’, by R. Chocron *et al.*, https://doi.org/10.1093/europace/euaf313**.

There is a paradox in the risk of sudden cardiac arrest (SCA) in individuals engaging in recreational and competitive sports. Physical activity will, in the long term, reduce cardiovascular morbidity and mortality. However, during vigorous physical activity, there is an acute, transient increase in the risk of cardiac arrhythmia and subsequent cardiac arrest.^1^ The growing popularity of endurance races such as half-marathons and marathons perfectly encapsulates this paradox. For the most part, participating in these activities will only have beneficial health effects. Conversely, for a small number of individuals who might have an underlying susceptibility, either in terms of a genetic vulnerability or occult heart disease, the same vigorous activity may trigger a ventricular arrhythmia and cardiac arrest.

In this issue of *Europace*, Chocron *et al*.^2^ describe the incidence, sex differences, and timing of SCA in endurance racing in Paris, France, over a 10-year period. The authors find that SCA during endurance racing is rare, but that there are certain common characteristics to these events.

The incidence of SCA in this study among endurance racing is 15.9 and 5.7 per million participants for men and women, respectively. Similarly, slightly lower estimates were published in a novel study of half-marathons and marathons in the USA.^3^ Both studies agree that the burden of SCA is substantially higher in male participants compared to females. Chocron *et al*. speculate that the underlying mechanisms of cardiac arrest in this population may include structural remodelling of the heart and hormonal factors influencing arrhythmogenesis during physical activity. Further, the authors discuss whether this could be attributed to both acceleration and deceleration in the last phase of the race. Acceleration acts as a trigger for ventricular arrhythmia due to a surge in catecholamines, and deceleration triggers a large vagal reflex, leading to high levels of acetylcholine when catecholamine levels are already high.^2,4^ In this state, athletes can be more prone to re-entrant tachycardias and ventricular fibrillation.

A key limitation, however, is the absence of data on habitual training load. Endurance athletes can present a broad range of phenotypes, ranging from structurally normal hearts to idiopathic fibrosis to severe structural changes known as exercise-induced arrhythmogenic cardiomyopathy.^5^ Why some athletes develop fibrosis or marked remodelling while others do not, despite similar training volumes, remains uncertain.^5^ Even when phenotypes resemble inherited cardiomyopathy, their genetic backgrounds may differ fundamentally from those of the diseases they mimic.^5^ Given the rarity of SCA in athletes, understanding how chronic exercise exposure, structural adaptation, and genetic susceptibility interact remains challenging but essential.^4,5^

One of the most striking observations in the study is the timing of arrests. Participants in the 20 km marathon and half-marathon were 15× more likely to experience SCA in the final kilometre. In contrast, SCA in marathon runners was more evenly distributed along the course. Whether this difference reflects distinct participant populations or divergent pacing strategies between the two distances remains unknown. In addition, survival was remarkably high at 88% with excellent neurological outcomes. Universal witnessed arrests, immediate bystander cardiopulmonary resuscitation (CPR), and rapid defibrillation clearly contributed to these results. Survival from out-of-hospital cardiac arrest in the community is far lower, even under similarly favourable conditions.^6^ These findings provide strong justification for concentrating medical resources, automated external defibrillators (AEDs), and trained responders in the final stretch of endurance races, where risk appears to peak.

The clinical profiles of the affected individuals also offer important lessons. Curiously, 4 out of 15 (27%) cardiac arrest survivors had a family history of sudden cardiac death (SCD), underlining the importance of this anamnestic information. All survivors had normal left ventricular ejection fractions and very few conventional cardiovascular risk factors. After hospitalization, the aetiology of the cardiac arrest in the four cases with a family history of SCD was either unknown or non-ischaemic cardiomyopathy. Both aetiologies may have a genetic component that is important to uncover for the SCA victim and their families. Two endurance racers could not be resuscitated. However, an autopsy was not performed, and the cause of death is therefore not known. It is also unknown if these two cases of presumed SCD had a family history of SCD. If not, they might be the proband of an inherited cardiac disease in their family, and their surviving relatives may benefit from the results of a thorough post-mortem examination consisting of autopsy, toxicology, and genetics. In both cases, guidelines from the European Society of Cardiology on the prevention of SCD recommend a systematic autopsy in all such cases and the preservation of biological material for genetic analyses.^7^ Further, if the results of these investigations point towards an inherited cardiac disease, the guideline also provides a Class IB recommendation that first-degree relatives should be assessed in a specialized clinic.^7^ However, the process of assessing the risk of SCA in the surviving relatives in the context of genetic findings of unknown significance is a complicated process that requires expertise from the treating physician as well as shared decision-making with the patient. In summary, in both SCA survivors and SCD cases, the underlying cause of the cardiac arrest may have implications beyond the affected person. It should be investigated regardless of the outcome of CPR.

In summary, the work by Chocron *et al*. strengthens the evidence that SCA during endurance racing is rare and predominantly affects men, and the novel knowledge from this study is that SCA disproportionately occurs near the finish line. Overall, the findings provide reassurance to athletes and organizers while also highlighting opportunities to further enhance safety through targeted emergency preparedness and systematic post-event evaluation. As participation in endurance races continues to grow, our challenge is not to deter physical activity, which remains beneficial for population health, but to ensure safe endurance racing through optimal resuscitation, post-resuscitation care, and follow-up on potential inherited causes of SCA.

## Data Availability

NA.
